# Concerns and priority outcomes for children with advanced cancer and their families in the Middle East: A cross-national qualitative study

**DOI:** 10.3389/fonc.2023.1120990

**Published:** 2023-03-14

**Authors:** Sabah Boufkhed, Sema Yurduşen, Ghadeer Alarjeh, Fahad Ahmed, Waleed Alrjoub, Ping Guo, Sawsan Alajarmeh, Meltem Şengelen, Mustafa Cemaloğlu, Burça Aydın, Anwar Alnassan, Shireen Al-Awady, Tezer Kutluk, Omar Shamieh, Richard Harding

**Affiliations:** ^1^ Florence Nightingale Faculty of Nursing, Midwifery and Palliative Care, Cicely Saunders Institute, King’s College London, London, United Kingdom; ^2^ Humanitarian and Conflict Response Institute, The University of Manchester, Manchester, United Kingdom; ^3^ Department of Pediatric Oncology, Hacettepe University Faculty of Medicine and Cancer Institute, Ankara, Türkiye; ^4^ Ankara Bilim Üniversitesi, Department of Psychology, Ankara, Türkiye; ^5^ Center for Palliative and Cancer Care in Conflict, King Hussein Cancer Center, Amman, Jordan; ^6^ School of Nursing and Midwifery, Institute of Clinical Sciences, College of Medical and Dental Sciences, University of Birmingham, Birmingham, United Kingdom; ^7^ Department of Public Health, Hacettepe University Faculty of Medicine, Ankara, Türkiye; ^8^ Paediatric Palliative Care, King Hussein Cancer Center, Amman, Jordan; ^9^ Department of Palliative Care, King Hussein Cancer Center, Amman, Jordan; ^10^ Faculty of Medicine, The University of Jordan, Amman, Jordan

**Keywords:** palliative care, children, cancer, Middle East, Jordan, Turkey, conflict, pediatrics

## Abstract

**Introduction:**

Palliative care access is limited in the Middle-East, with few specialist centers and forcibly displaced migrants facing additional struggles to access care. Little is known about the specificities of providing palliative care to children and young people (CYP) with cancer. They are rarely asked directly their concerns and needs, which limits the provision of quality patient-centered care. Our study aims to identify the concerns and needs of CYP with advanced cancer and their families, in Jordan and Turkey.

**Method:**

A qualitative cross-national study in Jordan and Turkey with framework analysis was conducted two pediatric cancer centers in Jordan and Turkey. In each country, 25 CYP, 15 caregivers and 12 healthcare professionals participated (N=104). Most caregivers (70%) and healthcare professionals (75%) were women.

**Results:**

We identified five areas of concern: (1) Physical pain and other symptoms (e.g. mobility, fatigue); (2) Psychological concerns and needs (e.g. anger, psychological changes); (3) Spirituality, uncertainty over the future and use of “Tawakkul” (e.g. use of religion as a coping mechanism); (4) Negative impact on social life (e.g. social isolation, loss of support); (5) Burden on caregiver and their families (e.g. financial issues, siblings left behind). Psychological concerns were a priority for both CYPs and caregivers (particularly for refugee and displaced families) but often overlooked during routine care. CYP were able to share their own concerns and care priorities.

**Conclusions:**

Advanced cancer care must ensure assessment and management of concerns across the concerns identified. Developing child- and family-centered outcomes would ensure monitoring the quality of care. Spirituality had a more important role compared to similar investigation in other regions.

## 1 Introduction

The majority of children with palliative care needs (97%) live in low- and middle-income countries (LMIC) (1, 2). Cancer is the most common cause of serious health-related suffering at the end of life (3), and the Middle-East is predicted to have the highest increase of such suffering by 2060. Palliative care is an essential Universal Health Coverage service but relatively new in the region, with no countries reporting fully integrated palliative care within its health system (4, 5).

Data on symptoms and concerns of children and young people (CYP) with advanced illness are scarce. Methodological and ethical challenges of conducting primary research with this potentially vulnerable group have led to a lack of outcomes-focused research (6). A systematic review found that self-report primary data from CYP with advanced illness was rare (6, 7), and a subsequent study in Africa found it was feasible to conduct primary data collection with this population (7).

Little is known about the cultural specificities of pediatric palliative care in the Middle-East region. Further, the region has large-scale migration flux and hosts many refugees with advanced cancer facing additional challenges of compounded trauma and fragmented social support systems (8–10). A systematic review found that patients at the end of life and their families in Muslim-majority countries feel “selflessness” in their duties to family and caregivers, ambivalence towards being hopeful and hopeless, and strongly believe in an afterlife (11).

The lack of primary data collected with CYP in the Middle East is a key reason for the limited development of pediatric palliative care in the region (5). In 2018, Jordan had 11 million inhabitants, of whom 45% are aged under 18 (12), and Turkey had 82.3 million inhabitants and 28% of the population is under 18 (13).

It is crucial that evidence from adults is not used to drive quality care for children (7). The COVID epidemic has highlighted the pre-existing lack of investment in palliative care capacity, and challenges in ensuring culturally-appropriate decisions around treatment withdrawal (14).

Our study aimed to identify the palliative care symptoms and concerns of CYP with advanced cancer, and their families, in Jordan and Turkey.

## 2 Methods

### 2.1 Study design

This qualitative cross-sectional study adhered to COREQ reporting guidelines. It was framed within pragmatic epistemology to inform clinical practice (15). The local research teams SY, MS, GA, WA, SA (psychologist involved in care provision (n=1), researcher (n=1), and researchers and palliative care nurses not connected to the interviewees (n=3); male (n=1) and female (n=4) conducted in-depth semi-structured interviews.

### 2.2 Setting

The sites (one in each of Turkey and Jordan) provide multidisciplinary paediatric palliative care within relatively large and diverse populations including refugees and displaced people (16).

### 2.3 Sampling and recruitment

#### 2.3.1 Inclusion and exclusion criteria

We sampled three stakeholder populations. First, CYP aged 5 to 17 living with advanced cancer (stage III or IV), and seen by the palliative care team for at least one consultation at either study site. Second, an adult parent or caregiver (17) responsible for the care needs of a child below 18 who met the inclusion criteria above. Third, palliative care staff (medicine, nursing, social work, psychology or allied health professional) who had been providing paediatric palliative care for at least 6 months.

The following exclusion criteria were used:

- CYP unable to communicate their views or wishes via self-report during an in-depth interview or with the support of their caregiver, or via “draw & talk” and play methods; those speaking a language not supported by the study sites (Arabic, Turkish and English); currently enrolled in another study; deemed unable to give assent by their treating clinician.
- Caregivers deemed clinically unable to give consent by their child’s treating clinician.
- Staff with less than 6 months experience of clinical paediatric palliative care.

#### 2.3.2 Sampling

The estimated sample size per study site to achieve maximum variation and reach data saturation was: CYP n=25; parents or caregivers (hereafter referred to as “caregivers”) n=15; palliative care staff n=12. We purposively sampled on the following CYP characteristics: primary malignancy, gender, age and communication difficulty, and country of birth. For the caregivers, we purposively sampled by age, gender and relationship to patient, and country of birth, and for the HCP by age, gender, years of experience and profession.

#### 2.3.3 Recruitment

Clinicians identified eligible participants during weekly multidisciplinary team meetings, then discussed the study with the family at their subsequent clinical appointment. Those who expressed an interest were directed to the research team who shared the child age-specific and the caregiver information sheets, and addressed any further questions. In Jordan, all the eligible participants approached by the research team accepted to participate. In Turkey, 103 patients were identified eligible, and 53 agreed to participate.

### 2.4 Data collection

Interviews were conducted face-to-face at a quiet convenient place (e.g. empty clinic, meeting room) and audio recorded by researchers (SY, GA, WA, SA and MS), from 21 March 2019 to 08 January 2020 in Turkey and 23 April 2019 to 29 July 2020 in Jordan. Study-specific training sessions were delivered with ongoing support (PG and RH, e.g. qualitative research methods, interview skills with children). Due to introduction of COVID-19 restrictions, five interviews in Jordan were conducted by telephone. 

Topic guides were developed for each stakeholder group, and for each child age grouping using appropriate language. Standard verbal probing, “draw and write” and the use of toys to express feelings were used. For children with communication difficulties, caregivers supported the child to express themselves. Children aged 16-17 with sufficient capacity could choose to be interviewed alone.

### 2.5 Data management and analysis

Audio recordings were transcribed verbatim, translated into English, reviewed by the researchers for quality check then imported into NVivo 12 Pro for analysis.

Collaborative analysis was conducted across the partner sites (UK, Jordan and Turkey) using framework analysis. (18) Five researchers (SB, SY, GA, FA and WA), including the three main interviewers, with different expertise (palliative care nursing, psychology, global public health, epidemiology) collaborated to optimise data analysis and interpretation. Firstly, they each familiarised themselves with the data and developed preliminary codes using three to five interviews randomly selected (at least one per stakeholder group). The lead analyst (SB) integrated the preliminary codes and identified similar emergent themes, and presented a preliminary joint framework to the cross-national analysis team. Regular online meetings were conducted to discuss and refine the framework, which was subsequently applied by the whole analysis team to five further transcripts each adding any new emergent codes. Ongoing team discussions refined the framework. The framework was then agreed and presented to the senior team (TK, OS, RH) for review and refinement, before being applied to the remaining dataset. The key themes were charted into a single framework matrix, with cross-national discussions to interpret findings.

### 2.6 Ethics

Informed assent was obtained from all child participants, with informed parental consent. Caregivers and healthcare professionals (HCP) gave informed consent. Ethical approval was obtained from King’s College London (ref: HR-18/19-8838); Hacettepe University (ref: 16969557-25 or GO 19/40) and KHCC (proposal No. 18 KHCC 162).

## 3 Results

### 3.1 Participants

We recruited 105 participants (52 in Jordan, 53 in Turkey). One interview could not be completed due to the interviewed patient’s distress. Therefore, a total of 104 interviews were analyzed. **Tables 1**, **2** report the sample’s characteristics (N=104). The median age of the 50 CYPs was 13 (IQR: 9.0-16.0). Caregivers (70%) and palliative care staff were mostly female (75%) (See **Supplementary Files** for the sample's characteristics by country).

**Table 1:** Participants’ age (N=104).

	Overall (N=104)			Jordan (N=52)			Turkey (N=52)		
	median	IQR	N	median	*IQR*	N	median	*IQR*	N
CYP	13	9-16	50	15.4	10-17	25	11.5	9-14	25
Caregiver	35	29-36	30	35	26-41	15	36	32-38	15
HCP	32	31-39	24	32	27-35	12	32	31-41	12

CYP, Children and Young People; HCP, Healthcare Professional.

**Table 2:** Participants’ characteristics (N=104).

	CYP (N=50)		Caregiver (N=30)		HCP (N=24)	
	n	%	n	%	n	%
Gender:						
Female	27	*54*	21	*70*	18	*75*
Male	23	*46*	9	*30*	6	*25*
Country of birth:						
Azerbaijan	1	*2*	1	*3*	–	*-*
Jordan	21	*42*	10	*33*	–	*-*
Libya	1	*2*	2	*7*	–	*-*
Palestine	1	*2*	2	*7*	–	*-*
Syria	2	*4*	1	*3*	–	*-*
Turkey	24	*48*	14	*47*	–	*-*
Child’s diagnosis classification*						
I. Leukemias, myeloproliferative diseases, and myelodysplastic diseases	6	*12*	5	*17*	–	*-*
II. Lymphomas and reticuloendothelial neoplasms	13	*26*	5	*17*	–	*-*
III. CNS and miscellaneous intracranial and intraspinal neoplasms	2	*4*	4	*13*	–	*-*
IV. Neuroblastoma and other peripheral nervous cell tumors	–	*-*	10	*33*	–	*-*
IX. Soft tissue and other extraosseous sarcomas	5	*10*	1	*3*	–	*-*
VI. Renal tumors	2	*4*	2	*7*	–	*-*
VII. Hepatic tumors	–	*-*	1	*3*	–	*-*
VIII. Malignant bone tumors	19	*38*	2	*7*	–	*-*
X. Germ cell tumors, trophoblastic tumors, and neoplasms of gonads	2	*4*	–	*-*	–	*-*
XI. Other malignant epithelial neoplasms and malignant melanomas	1	*2*	–	*-*	–	*-*
Relationship to child:						
Mother	–	*-*	20	*67*	–	*-*
Father	–	*-*	9	*30*	–	*-*
Grandmother	–	*-*	1	*3*	–	*-*
Healthcare profession:						
Medical (nurse, oncologist)	–	*-*	–	*-*	16	*67*
Non-medical**	–	*-*	–	*-*	8	*33*

*Classification using the International Classification of Childhood Cancer (ICCC) 3rd edition. Main Classification Table from the ICCC-3 based on ICD-O-3. Available at: https://seer.cancer.gov/iccc/iccc3.html.

**Non-medical professions include: for Jordan: Child Life specialist (n=3); Psychosocial Consultant (n=1); Social Worker (n=1); for Turkey: Nutritionist (n=2); Ergotherapist (n=1).

CYP, Children and Young People; HCP, Healthcare Professional.

### 3.2 Overview of key concerns and needs

The analysis revealed five key domains of concerns and priorities: (1) Physical pain and symptoms; (2) Psychological concerns and needs; (3) Spirituality, uncertainty over the future and use of “Tawakkul”; (4) Negative impact on social life; and (5) Burden on caregiver and their families (see **Table 3**).

**Table 3:** Key concerns and priorities for children and young people with advanced cancer, their families and palliative care providers in Jordan and Turkey.

Key themes	Sub-themes
**1. Physical pain and symptoms**	Symptoms or concerns related to physical health.
	i. Pain
	ii. Other physical signs and symptoms (e.g. mobility, fatigue)
**2. Psychological concerns and needs**	Psychological wellbeing.
	i. Psychological signs and symptoms:
	- *Anger and emotional distress*
	- *Changes in mood*
	ii. Psychological concerns and needs:
	- *CYP and caregivers need to talk, and their issues to be heard and addressed*
	- *Need reassurance*
	- *Need for professional psychological support*
	iii. Importance of sharing and gathering experience throughout the illness journey:
	- *Own experience: Importance for CYP and caregivers to build their own experience of, for example, hospitalization and symptoms*
	- *Other people’s experience: Importance for CYP and caregivers to hear about other people’s experience to normalize their own experience*
**3. Spirituality, uncertainty over the future and use of “Tawakkul”**	Spiritual concerns, sources of uncertainty over the future faced by CYP and caregivers and use of religious coping.
	i. “Tawakkul”, faith and reliance on God ii. Hopeful/Hopelessness
	iii. Uncertainties over the future related to child’s disease, child’s immediate and longer-term future can cause anxiety and existential concerns.
**4. Negative impact on social life**	Detrimental effect on social life of patients and their families, such as activities, relationship, and interactions with the broader society.
	i. Social isolation
	ii. Need to social support from extended family and beyond
	iii. Importance for the child to play and go to school
**5. Burden on caregivers and their families**	Burden on families’ personal life and social function, including siblings
	i. Caregivers’ life and work are shattered
	ii. Caring for and addressing other sibling’s needs
	iii. Financial burden (direct and indirect economic costs affecting families, and the mechanisms that are needed to access appropriate care in practice)

#### 3.2.1 Physical pain and symptoms

Pain was by far the most important concern reported by participants, including procedural pain (e.g. fear of needles and invasive procedures) but also disease-related pain that had profound effects.

*“The most prominent thing was the pain and my appearance, like I was always in pain, always, always in pain. My appearance changed. I didn’t recognize myself” - Jordanian male CYP aged [11-16]*

Painful treatments gave children negative associations with HCPs and hospital, and children particularly feared needles

*“When you enter the hospital you’re not in pain, but when they give you chemotherapy the exhaustion starts and the nausea and the vomiting and more than one things [ … ] I’ll never do what the doctors say again. It always hurts” Turkish male CYP aged [05-07]*

*“She was scared of the nurses, anyone dressed in blue made her shake. At first, she would start shaking whenever she saw a nurse, she was scared of needles.” Jordanian mother aged [31-35]*

Pain was described as a major barrier to children performing their daily and leisure activities

*“M: I also can’t eat most things. I have a stomach ache when I eat.” Turkish male CYP aged [08-10]*

A child explained that the pain made her think about the end of life

*“Patient: After the operation, I never expected the operation like this. I had a lot of pain, and I could never move my foot. I said, it’s over now, that’s it. Mean it is fate. This fate.*

*Interviewer: What was it fate?*

*Patient: I don’t know [laughs], I guess that moment…. I mean something like the end of life, at that moment it was really hard for me [ … ] My world is almost finished. [laughs]” Turkish female CYP aged [11-16]*

HCPs related this back to the importance of pain relief to enable the child to mobilize and undertake independent function.

*“But the most important point is controlling the pain and for the child to have the ability to move or to reach the bathroom, to move. This is one of the most uncomfortable things for the child and his parents” Medical HCP female in Jordan aged [46-50]*

Additionally, common reported physical symptoms were fatigue, weakness/numbness, nausea, vomiting, constipation, fever, swelling of a body part, sleep issues, headache, loss of appetite, and a few reported breathing difficulties. Changes in physical appearance (e.g. deformity, hair loss, yellow in the eyes, weight change) had a serious impact on mental health.

Mental health issues may also have a physical impact on the child

*“Some people have diarrhea or constipation, but constipation and diarrhea are not just protocols of treatment, some people get them from fear, some people get numbness in their ends and headaches as well from overthinking not just from chemotherapy and radiation therapy.” Non-medical HCP female in Jordan aged [26-30]*

Conversely, HCPs also reported a psychological impact of the child’s physical problems

*“There was a patient that I will never forget, it was really sad, she had met all over her body, she had rhabdomyosarcoma but it was very dysmorphic, her face, her hands, everything was different, there were masses all over, I have never seen such a thing. She first came three years ago and I had just recently started working here, and this girl, [T], I will never forget her, she went through a lot, she was so tough to look at, even her siblings couldn’t come and see her.” Medical HCP male in Jordan aged [31-35]*

#### 3.2.2 Psychological concerns and needs

The majority of CYPs and caregivers identified psychological concerns and needs as their major concern.

*“Psychology is 50% of the treatment” Jordanian male CYP aged [11-16]*

These needs were often seen as not addressed by HCP, who concurred that physical care was their priority despite recognizing psychological needs’ importance. Anger and irritability were common emotions reported by caregivers and especially in CYPs, who were also concerned about boredom. Changes in CYP’s behaviors seemed to be indicators helping to monitor their physical and mental health. HCPs also described anger and aggression reactions.

*“So kids who are less than 3 years for example, they feel pain but it’s mostly severe exhaustion that they experience and they can’t move and some of them become angry in a way and they can’t handle anyone, and from 3 to 5 years old, they also become very angry as well. They hit their siblings and some of them might use a lot of profanity in their speech” Medical HCP female in Jordan aged [46-50]*

This was compounded for migrant children

*“We stayed at the border for three days, we slept in the middle of the desert. I thought he was scared of the police or the shootings, and he was stuck to me the entire time. When I got off to open the suitcase at the checkpoint, he would scream his head off, I thought he was scared. He has been normal his entire life, he never complained.” Palestinian mother receiving care in Jordan aged [31-35]*

Both caregivers and CYPs’ interviews highlighted the need to talk through their experiences and to receive professional psychological support. Almost as importantly, caregivers and CYPs expressed the need for their issues *to be heard* and addressed by HCP. CYPs, and in particular caregivers, needed reassurance from the HCP.

Further, most of the CYP, especially the oldest, wanted to be informed about their diagnosis but were often excluded from discussions about their health. A few shared that they were glad their parents concealed negative information.

#### 3.2.3 Spirituality, uncertainty over the future and use of “Tawakkul”

Faith and reliance on God were a predominant concept identified as “tawakkul” (10). Religious belief enabled coping with uncertainty and keeping hope for the future. Most interviews referred to expressions such as “Thank God” or to putting their fate “in God’s hands”.

*“Hope Allah cures everybody and us too. If only God heals our child, as long as the child is with me, I can accept to be beggar from door to door [which means she accepts to be poor].” Turkish grandmother aged [51-55]*

Caregivers reported performing religious rituals with the child when s/he had uncontrolled pain, and several reported that they increased their religious practice after the diagnosis.

*“We don’t say anything but just when it’s the right moment, we tell him that the same thing all the time, because we are the believers. This is a test world, we will pass this test. I mean the God made us that, examined us, we will pass it, we will get out of here with God willing” Turkish father aged [36-40]*

A CYP highlighted that she stopped praying due to the illness although her spirituality was very important

*“Sometimes people are too sick to wash up for prayer, or in so much pain, or too upset to pray” Jordanian female CYP aged [11-16]*

Religion also shaped the understanding of diagnosis

*“I am confused, some people say I am possessed, some people come and read the Quran for me. They say I am envied* ^1^ *and bring over Sheikhs.” Jordanian female CYP aged [08-10]*

A sense of acceptance of the disease as part of life also supported coping

*“I don’t worry so much anymore. Because all rivers fall into the sea. No matter how much I am worried, whatever will be, will be. There is no more than that. Because, when we are worried, this place becomes more unbearable.” Syrian girl patient receiving care in Jordan aged [17-18]*

A CYP advised peers to find peace by accepting the situation and socializing in the hospital:

*“In this process, iiiii* ^2^ *when I first came here, I had a hard time. So, I had a lot of difficulties before I had surgery. I can even give some advice, I was never with peace here [laughs]. I was hating this hospital. I tried to get used to a bit after the surgery. I made friends. So it’s easier. I can advise to be at peace with here. [laughs]” Turkish female CYP aged [11-16]*

The ability to keep or lose hope was an important feature of participants’ spirituality and shaped their emotional journey and ability to cope

*“Let me tell you clearly that, I have no hope. Because we’ve been here for 5 months, 10 children we’ve stayed with have passed away. This disease, the doctors … I mean they’re doing more than they can, but there’s nothing they can do.[ … ] I can’t get rid of the pain inside of me. Because there’s nothing to do.” Turkish father aged [41-45]*

Despite a reliance on prayer for a positive outcome, it was also difficult to remain hopeful over time:

*“I say, we’ll pray, we’ll get better, she says, “Mommy, we’ve been here for two years, I’m not getting better now.” I said such a thing cannot happen, you don’t know the future, and you just act as if she will recover. I said if you want to heal, you will struggle to get better. I always try to give her support, because we, as a family, my husband, my children, my mother, father, sister, the whole family, the road gets longer, the less our patience and the heavier our burden, we fall into pessimism. We say everything is in vain.” Turkish mother aged [36-40]*

HCP reported hesitance in discussing spirituality and end of life with CYP

*“It’s hard to discuss spiritual topics with children because it’s a sensitive topic and it may emotionally provoke them. [ … ] The problem is that for some time, we wouldn’t frankly talk about death in our sittings, especially if the kids were present; we wouldn’t bring up spiritual topics much like what will happen after they die. Maybe we’d talk to the parents more about it when their child isn’t present. We’d tell them that if their child passes away, God will replace their losses or that God will choose the best fate. It’s hard to discuss spiritual topics with children because it’s a sensitive topic and it may emotionally provoke them.” Medical HCP female in Jordan aged [31-35]*

Uncertainty over the future was one of the major concerns of CYPs and led to existential questioning and hopelessness. Most CYP, and some caregivers, were worried about treatment duration and outcomes like their ability to walk, go back to school or play, i.e. to go back to normal.

*“I feel sad. Then, when it is over, I wonder how I will keep up with things? The other operation forced me a lot. Now, because of the outpatient treatment, my walking distress, though it has decreased but there is a little bit. I can’t walk like every other person, I’m getting tired anyway. Uh, it pushed me hard a little bit, how do things will be going when I start school. I wonder how I will keep up with a…. fast-paced life, the tempo, when the school starts, home work, exams etc. I am a little scared anyway, I have some worries” Turkish female CYP aged [11-16]*

Some, especially teenagers, were particularly worried about their future education

*“I am going to waste my future, the semester is almost over.” Jordanian female CYP [17-18]*

Few CYP worried about cancer stigma preventing employment and marriage prospects

*“I had a patient that told me that he won’t ever be able to work or get married because he’ll always be labelled as a cancer patient even if he gets better. This patient didn’t have a curative prognosis, but he still thought that even if he was ever cancer-free, he’d still be labelled as someone with cancer. He said that families would reject his marriage proposals just because he had cancer.” Non-medical HCP female in Jordan aged [26-30]*

For HCPs, CYP and caregivers described fear of death. They felt that younger children may not grasp the concept of death, but that realization may grow

*“There were 6 cycles, but the treatment is not finished. When we say let’s give 3 more, let’s give 5 more, that kid understood that this is not going straight. No matter what medicine you give. Then the child understands and fears death without saying that things are not going well.” Medical HCP female in Turkey aged [36-40]*

Some HCPs acknowledged that there was a lack of support for grieving and bereavement for caregivers after their children’s death. This may cause distress for caregivers, and HCP who may feel they abandon them in difficult times

*“someone once said to me “my wife and I are mentally destroyed” he even changed the room he was in, we even forget the families, we forget the families after being DNR, after the patient dying, our relationship with them gets cut off. “ Medical male HCP aged [31-35] in Jordan*

#### 3.2.4 Negative impact on social life

Social isolation was one of the biggest concerns expressed by CYPs and caregivers. Accessing cancer treatment often meant being far from the rest of family and social support. Infection control also caused physical separation. For caregivers (mostly mothers) additional duties such as physical care work (e.g. carrying or bathing the child), emotional labor (e.g. addressing children’s need for attention) and the fear of infection were the most reported reasons for social isolation.

Caregivers’ needs ranged from practical support to caring for siblings, to social interaction without mentioning the disease, and professional psychological support. Social isolation was particularly concerning for immigrants, refugees or those who had travelled far for treatment.

*“We just come out and walk around. We’re not seeing anyone. In fact, this is the most important, we are going through an unsocial process.” Turkish mother aged [36-40]*

*“In Gaza I am with my family, but here I am not.” Palestinian female CYP receiving care in Jordan aged [08-10]*

It was a key concern that children could not play with or see friends or go to school because of pain, symptoms, or fear of infection. They missed “hanging out” with friends and family outdoors although they used technology to remain connected with loved ones or to fight boredom.

Finally, participants reported concerns about the lack of awareness about cancer in the society that could lead to stigma and gossip

*“people outside look at me pityingly, look at your child pityingly. They should not look like that. No one should look at anyone pityingly [ … ] people should not say, do not come close to this child otherwise you will get it too, saying ahh, saying aww saying thhuu … Or they shouldn’t say why you don’t have hair. Or they shouldn’t say why you are wearing a mask.” Turkish mother aged [18-25]*

#### 3.2.5 Burden on caregivers and their families

Caregivers were distressed by not being able to be with all their children, who were often taken care of by other women of the family like an aunt or grandmother. Mothers, in particular, reported concerns about leaving other children behind.

*“My mother and father [took care of them]. My daughter was in kindergarten, she would go to her aunt’s, her aunt has kids around her age, and my son would stay with my mother. I went through very tough times, the last year was very tough.” Jordanian mother aged [31-35]*

Most of interviews with caregivers and HCP described caregivers’ emotional distress

*“As we see the suffering of children, we are ruined. [ … ] I do not think that any mother can survive with this feeling. [Sighed]” Turkish mother aged [26-30]*

A major concern in caregivers’ interviews and corroborated in HCP’s were the indirect costs and logistical challenges in accessing care far from home.

*“Their father works in the city council, he is a cleaner, and I spend most of my time in the hospital. We have a hard time with transportation, we call a bus to drive us around. The bus costs me 30 JD. Yesterday she had an appointment with Dr. [name], he said she is not to be admitted, and told us to go back home and come back the next day. I told him I couldn’t afford it, I can’t pay 30 JD twice in two days, my husband only gets paid 228 JD, he works on a daily-basis payment and he’s not fixed. I told them I would stay in the ER till the next day, and once a room is available I’ll transfer to it.” Jordanian mother aged [46-50]*

Several caregivers reported they had to take unpaid days off work to accompany the child to hospital, and worried about losing income or getting fired. Some mothers had to stop working **care for the child.**

*“I’d go to work with my heart on fire. I wait and count the hours and wonder when I’m going home, when I can retire. If they don’t deduct from our salaries, I would leave work and sit at home with them. I have no relationships, I don’t even see my neighbor. I just go to my Caregivers’ house and come back. So honestly, I have dedicated all of my time to the children” Jordanian mother aged [41-45]*

The interviews revealed that refugees and foreign patients had additional financial barriers related to currency difference and transport.

*“Like I spent 3 Dinars on a taxi, that’s 15 Libyan Dinars. You understand? It’s like the 3000 Jordanian Dinars I exchanged over 3 or 4 days, 17000 Libyan Dinar. Three or four days I’m not talking about a treatment trip, you understand? Like I went and bought 10 Dinars worth of fruit or I don’t know what a meal, multiply that by 5.” Libyan father in Jordan aged [41-45]*

Additional direct costs for refugees and foreign patients included hospital and treatment fees, met from various sources including family or co-workers.

## 4 Discussion

### 4.1 Main findings and comparison with existing literature

Our data reveal five dominant domains of concern among CYP with advanced cancer and their families in Jordan and Turkey. These substantive findings add new insight to the small self-report evidence base for this population. Our findings highlight the importance of pain management for CYP, and the urgent need to improve access to psychosocial support for patients and families, with particular attention to refugee and foreign patients. Pain management, therefore, need to embrace the “total pain” concept (19) to improve palliative care provision.

Concurring with other research on cancer experiences in the region, we identified faith and reliance on God (tawakkul) as an important coping mechanism for families and patients that helps overcoming fear of death and end-of-life (8, 10, 20). Tawakkul is an important concept for Muslim patients. 97% of the population in Jordan and 99% in Turkey are Muslim (21, 22). However, there is no consensus yet on what spiritual needs are and little is known about whether or how religion could have a positive or negative impact on mental health (23). The prior systematic review of symptoms and concerns among CYP with advanced illness reported a dearth of evidence in the spiritual domain (24). Within our study, CYPs did not discuss their fear of dying directly or fear of going to sleep. Fear of death was mostly reported by HCPs and rarely by caregivers, which may be related to the cultural context and a need to hold on to ‘tawakkul’.

Our data suggests that CYP tend to be excluded from discussions about their own health despite willingness to be informed. While family-based models encourage shared decision-making, it is not widely spread in pediatric care (25) and in the Middle East (26). Further, the limited evidence about shared decision-making models’ effectiveness on patient outcomes and its focus on Western countries (27–29) calls for further investigation on existing shared-decision models in the region.

### 4.2 Implications for practice

Our findings support a family-centered care approach for pediatric palliative care in Jordan and Turkey. It is in line with other studies describing the central place of extended families in care, and its importance culturally as suggested in other research in Muslim-majority countries and in life-threatening illness among adults in the region (8, 11, 30). The isolation of the children and their caregivers, far from people providing them with social support, was an important source of distress, which was amplified for migrant and refugee patients (9).

Our findings demonstrate the urgent need to address the financial and psychosocial burden faced by caregivers, especially for foreign and refugee patients and those with limited resources. A 2021 systematic review of the burden of out-of-pocket expenditures faced by patients diagnosed with cancer and their caregivers in LMIC showed the significant out-of-pocket costs related to treatment “most of which is spent on cancer medications, followed by caregiver expenses, and transport and travel expenses” (31). However, no study was identified from the Middle-East region.

### 4.3 Strengths and limitations

Our study has successfully recruited and interviewed CYP with advanced cancer, which has been an omission in studies in this population generally, and has not been conducted previously in this region (24). It provides multistakeholder perspectives on the themes, and included experiences of refugees. Our innovative collaborative approach to data analysis strengthened culturally and contextually relevant identification and interpretation of findings. The analysis was, however, time-intensive and demanding.

Our research also presents some limitations. Given the limited availability of palliative care research and practice in the region and the difficulty of conducting such research, we only focused on cancer and included one site per country, which are regional leaders of palliative care. The needs identified may be therefore underestimated and may not be generalizable to the whole region. The care offered in both centers and countries were overall similar but had some variation for non-medical services. This led researchers to sometimes emphasize issues that may be locally relevant. Finally, collecting data among children, especially on a sensitive topic, and with caregivers was challenging in terms of both communication with the interviewees, and emotional impact on the research team.

### 4.4 Conclusion

Our study described what matters for children and young people with advanced cancer and their families in the Middle East. Outcome measures can improve pediatric palliative care by ensuring that the care addresses the needs of patients and their families (27). This study provides the primary data to develop child- and family-centered assessment and outcome measurement that reflect what matters (1). Pain needs to be considered holistically by palliative care professionals so as to address CYP and their caregivers’ significant psychosocial support needs. The additional high emotional, social and financial burden faced by caregivers, recognized by their HCPs, calls for a sustainable investment in palliative care in line with Universal Health Coverage to better support patients and families.

## Data availability statement

The datasets presented in this article are not readily available because the data are qualitative and we cannot share the transcripts. Requests to access the datasets should be directed to SB, sabah.boufkhed@manchester.ac.uk.

## Ethics statement

Ethical approval was obtained from King’s College London (ref: HR-18/19-8838); Hacettepe University (ref: 16969557-25 or GO 19/40) and KHCC (proposal No. 18 KHCC 162). Written informed consent to participate in this study was provided by the participants’ legal guardian/next of kin.

## Author contributions

PG, RH, OS, and TK contributed to conception and design of the study. SY, GA, WA, FA, SA and MS collected the data. SB organized and managed the data, and led and coordinated the cross national. SB, SY, GA, FA, and WA analyzed and interpreted the data. SB wrote the first draft of the manuscript. All authors contributed to the article and approved the submitted version.

## Footnotes

1 in this context, this refers to harmful envy [hasad] and sending him the ‘evil eye’ [al ‘ayn]

2 Turkish equivalent for “mmmmm”
