# Supplementary material for: Concerns and priority outcomes for children with advanced cancer and their families in the Middle East: A cross-national qualitative study
Source: Front Oncol. 2023 Mar 14;13:1120990. doi: 10.3389/fonc.2023.1120990 (PMC10043430; doi:10.3389/fonc.2023.1120990)
Supplement: Supplementary file 1 [file Table_1.docx]

Supplementary Material

Concerns and priority outcomes for children with advanced cancer and their families in the Middle East: a cross-national qualitative study

**Sabah Boufkhed^1,2*^, Sema Yurduşen^3^, Ghadeer Alarjeh^4^, Fahad Ahmed^3^, Waleed Alrjoub^4^, Ping Guo^1,5^, Sawsan Alajarmeh^4^, Meltem Sengelen^6^, Mustafa Cemaloğlu^3^, Burça Aydın^3^, Anwar Alnassan^7^, Shireen Al-Awady^7^, Tezer Kutluk^3^, Omar Shamieh^4,8, 9^, Richard Harding^1^**

*** Correspondence:** Dr Sabah Boufkhed: [sabah.boufkhed@manchester.ac.uk](mailto:sabah.boufkhed@manchester.ac.uk)

# Supplementary Tables

Supplementary table 1. Participants’ age per country (N=104)

|  | **Overall**  (N=104) | | | **Jordan**  (N=52) | | | **Turkey**  (N=52) | | |
| --- | --- | --- | --- | --- | --- | --- | --- | --- | --- |
|  | **median** | **IQR** | **N** | **median** | ***IQR*** | **N** | **median** | ***IQR*** | **N** |
| CYP | 13 | 9-16 | 50 | 15.4 | 10-17 | 25 | 11.5 | 9-14 | 25 |
| Caregiver | 35 | 29-36 | 30 | 35 | 26-41 | 15 | 36 | 32-38 | 15 |
| HCP | 32 | 31-39 | 24 | 32 | 27-35 | 12 | 32 | 31-41 | 12 |

Note: CYP: Children and Young People; HCP: Healthcare Professional

Supplementary table 2. Participants’ characteristics per country (N=104)

|  | **Overall**  (N=104) | | | | | | **Jordan**  (N=52) | | | | | | **Turkey**  (N=52) | | | | | | |
| --- | --- | --- | --- | --- | --- | --- | --- | --- | --- | --- | --- | --- | --- | --- | --- | --- | --- | --- | --- |
|  | **CYP** | | **Caregiver** | | **HCP** | | **CYP** | | **Caregiver** | | **HCP** | | **CYP** | | **Caregiver** | | **HCP** | | |
|  | (N=50) | | (N=30) | | (N=24) | | (N=25) | | (N=30) | | (N=24) | | (N=25) | | (N=30) | | (N=24) | | |
|  | **n** | ***%*** | **n** | ***%*** | **n** | ***%*** |  | ***n*** | **%** | ***n*** | **%** | ***n*** | **n** | ***%*** | **n** | ***%*** | **n** | ***%*** |  |
| **Gender:** |  |  |  |  |  |  |  |  |  |  |  |  |  |  |  |  |  |  |  |
| Female | 27 | *54* | 21 | *70* | 18 | *75* | 14 | *56* | 12 | *80* | 9 | *60* | 13 | *52* | 9 | *60* | 9 | *60* |  |
| Male | 23 | *46* | 9 | *30* | 6 | *25* | 11 | *44* | 3 | *20* | 3 | *20* | 12 | *48* | 6 | *40* | 3 | *20* |  |
| **Country of birth:** |  |  |  |  |  |  |  |  |  |  |  |  |  |  |  | *0* |  |  |  |
| Azerbaijan | 1 | *2* | 1 | *3* | - | *-* | - | *-* | - | *-* | - | *-* | 1 | *4* | 1 | *7* | - | *-* |  |
| Jordan | 21 | *42* | 10 | *33* | - | *-* | 21 | *84* | 10 | *67* | - | *-* | - | *-* | - | *-* | - | *-* |  |
| Libya | 1 | *2* | 2 | *7* | - | *-* | 1 | *4* | 2 | *13* | - | *-* | - | *-* | - | *-* | - | *-* |  |
| Palestine | 1 | *2* | 2 | *7* | - | *-* | 1 | *4* | 2 | *13* | - | *-* | - | *-* | - | *-* | - | *-* |  |
| Syria | 2 | *4* | 1 | *3* | - | *-* | 2 | *8* | 1 | *7* | - | *-* | - | *-* | - | *-* | - | *-* |  |
| Turkey | 24 | *48* | 14 | *47* | - | *-* | - | *-* | - | *-* | - | *-* | 24 | *96* | 14 | *93* | - | *-* |  |
| **Child's diagnosis classification*** |  |  |  |  |  |  |  |  |  |  |  |  |  |  |  |  |  |  |  |
| I. Leukemias, myeloproliferative diseases, and myelodysplastic diseases | 6 | *12* | 5 | *17* | - | *-* | 6 | *24* | 5 | *33* | - | *-* | - | *-* | - | *-* | - | *-* |  |
| II. Lymphomas and reticuloendothelial neoplasms | 13 | *26* | 5 | *17* | - | *-* | 2 | *8* | 1 | *7* | - | *-* | 11 | *44* | 4 | *27* | - | *-* |  |
| III. CNS and miscellaneous intracranial and intraspinal neoplasms | 2 | *4* | 4 | *13* | - | *-* | - | *-* | 3 | *20* | - | *-* | 2 | *8* | 1 | *7* | - | *-* |  |
| IV. Neuroblastoma and other peripheral nervous cell tumors | - | *-* | 10 | *33* | - | *-* | - | *-* | 4 | *27* | - | *-* | - | *-* | 6 | *40* | - | *-* |  |
| IX. Soft tissue and other extraosseous sarcomas | 5 | *10* | 1 | *3* | - | *-* | 3 | *12* | 1 | *7* | - | *-* | 2 | *8* | - | *-* | - | *-* |  |
| VI. Renal tumors | 2 | *4* | 2 | *7* | - | *-* | 1 | *4* | 1 | *7* | - | *-* | 1 | *4* | 1 | *7* | - | *-* |  |
| VII. Hepatic tumors | - | *-* | 1 | *3* | - | *-* | - | *-* | - | *-* | - | *-* | - | *-* | 1 | *7* | - | *-* |  |
| VIII. Malignant bone tumors | 19 | *38* | 2 | *7* | - | *-* | 12 | *48* | - | *-* | - | *-* | 7 | *28* | 2 | *13* | - | *-* |  |
| X. Germ cell tumors, trophoblastic tumors, and neoplasms of gonads | 2 | *4* | - | *-* | - | *-* | - | *-* | - | *-* | - | *-* | 2 | *8* | - | *-* | - | *-* |  |
| XI. Other malignant epithelial neoplasms and malignant melanomas | 1 | *2* | - | *-* | - | *-* | 1 | *4* | - | *-* | - | *-* | - | *-* | - | *-* | - | *-* |  |
| **Relationship to child:** |  |  |  |  |  |  |  |  |  |  |  |  |  |  |  |  |  |  |  |
| Mother | - | *-* | 20 | *67* | - | *-* | - | *-* | 12 | *80* | - | *-* | - | *-* | 8 | *53* | - | *-* |  |
| Father | - | *-* | 9 | *30* | - | *-* | - | *-* | 3 | *20* | - | *-* | - | *-* | 6 | *40* | - | *-* |  |
| Grandmother | - | *-* | 1 | *3* | - | *-* | - | *-* |  |  | - | *-* | - | *-* | 1 | *7* | - | *-* |  |
| **Healthcare profession:** |  |  |  |  |  |  |  |  |  |  |  |  |  |  |  |  |  |  |  |
| Medical (nurse, oncologist) | - | *-* | - | *-* | 16 | *67* | - | *-* | - | *-* | 7 | *58* | - | *-* | - | *-* | 9 | *75* |  |
| Non-medical** | - | *-* | - | *-* | 8 | *33* | - | *-* | - | *-* | 5 | *42* | - | *-* | - | *-* | 3 | *25* |  |

Notes:

* Classification using the International Classification of Childhood Cancer (ICCC) 3rd edition. Main Classification Table from the ICCC-3 based on ICD-O-3. Available at: https://seer.cancer.gov/iccc/iccc3.html

** Non-medical professions include: for Jordan: Child Life specialist (n=3); Psychosocial Consultant (n=1); Social Worker (n=1); for Turkey: Nutritionist (n=2); Ergotherapist (n=1)

CYP: Children and Young People; HCP: Healthcare Professional
